# Identification of key molecules in COVID-19 patients significantly correlated with clinical outcomes by analyzing transcriptomic data

**DOI:** 10.3389/fimmu.2022.930866

**Published:** 2022-08-22

**Authors:** Zehua Dong, Qiyu Yan, Wenxiu Cao, Zhixian Liu, Xiaosheng Wang

**Affiliations:** ^1^ Biomedical Informatics Research Lab, School of Basic Medicine and Clinical Pharmacy, China Pharmaceutical University, Nanjing, China; ^2^ Big Data Research Institute, China Pharmaceutical University, Nanjing, China; ^3^ Jiangsu Cancer Hospital, Jiangsu Institute of Cancer Research, The Affiliated Cancer Hospital of Nanjing Medical University, Nanjing, China

**Keywords:** COVID-19, intensive care unit, mechanical ventilatory support, viral loads, antiviral immune responses, COVID-19 clusters

## Abstract

**Background:**

Although several key molecules have been identified to modulate SARS-CoV-2 invasion of human host cells, the molecules correlated with outcomes in COVID-19 caused by SARS-CoV-2 infection remain insufficiently explored.

**Methods:**

This study analyzed three RNA-Seq gene expression profiling datasets for COVID-19 and identified differentially expressed genes (DEGs) between COVID-19 patients and normal people, commonly in the three datasets. Furthermore, this study explored the correlation between the expression of these genes and clinical features in COVID-19 patients.

**Results:**

This analysis identified 13 genes significantly upregulated in COVID-19 patients’ leukocyte and SARS-CoV-2-infected nasopharyngeal tissue compared to normal tissue. These genes included *OAS1*, *OAS2*, *OAS3*, *OASL*, *HERC6*, *SERPING1*, *IFI6*, *IFI44*, *IFI44L*, *CMPK2*, *RSAD2*, *EPSTI1*, and *CXCL10*, all of which are involved in antiviral immune regulation. We found that these genes’ downregulation was associated with worse clinical outcomes in COVID-19 patients, such as intensive care unit (ICU) admission, mechanical ventilatory support (MVS) requirement, elevated D-dimer levels, and increased viral loads. Furthermore, this analysis identified two COVID-19 clusters based on the expression profiles of the 13 genes, termed COV-C1 and COV-C2. Compared with COV-C1, COV-C2 more highly expressed the 13 genes, had stronger antiviral immune responses, were younger, and displayed more favorable clinical outcomes.

**Conclusions:**

A strong antiviral immune response is essential in reducing severity of COVID-19.

## Background

The coronavirus disease 2019 (COVID-19) pandemic caused by severe acute respiratory syndrome coronavirus 2 (SARS-CoV-2) infection has caused nearly 477 million cases and more than 6 million deaths as of March 25, 2022 (1). Moreover, the number of COVID-19 cases have been dramatically increasing since the virus has evolved to higher infectivity, such as the Omicron variant (2). Some key molecules contributing to SARS-CoV-2 invasion of human host cells have been identified, including angiotensin-converting enzyme 2 (ACE2), transmembrane serine protease 2 (TMPRSS2), FURIN, cathepsins B/L (CTSB/L), cyclin G associated kinase (GAK), AP2 associated kinase 1 (AAK1), and two pore segment channel 2 (TPCN2) (3–8). Besides, myriad studies have explored the factors responsible for the significantly different clinical outcomes in COVID-19 patients, including age (9), gender (10), viral load (11), immune (12), nutrient intake (13), and underlying medical conditions (14).

Although multi-omics analyses have identified molecular characteristics significantly associated with COVID-19 outcomes (15–17), there exist discrepant results among different studies. To overcome this limitation, in this study, we identified differentially expressed genes (DEGs) between COVID-19 patients and normal people in three datasets, respectively, and uncovered common DEGs among them. We further analyzed associations between these genes and clinical features of COVID-19 patients. This study is expected to provide molecular insights into mechanisms of COVID-19 infection and severity.

## Methods

### Datasets

Three RNA-Seq gene expression profiling datasets for COVID-19 were downloaded from the NCBI Gene Expression Omnibus (GEO) (https://www.ncbi.nlm.nih.gov/geo/). These datasets included GSE157103 (17) (gene expression profiles in 100 COVID-19 patients’ leukocyte samples) and GSE152075 (11) and GSE156063 (18) (gene expression profiles in SARS-CoV-2-infected human tissues from nasopharyngeal swabs). **Table 1** is a summary of these datasets, and more details on these datasets are presented in the **Supplementary Table S1**.

**Table 1:** A summary of the datasets.

GSE157103	
**Tissue resource**	Leukocyte samples from hospitalized patients
**Sample size**	n = 128 (102 patients versus 26 controls)
**Demographic characteristics**	
Male sex – No. (%)	74 (57.8)
Age Range – No. (%)	
Younger than 50	31 (24.6)
51–60	21(16.7)
61–70	30 (23.8)
71–80	23 (18.3)
81 and older	20 (15.9)
Missing data	1 (0.8)
**Clinical characteristics**	
ICU admission – No. (%)	66 (52.4)
Hospital free days at 45 days	29.5 (median)
Mechanical ventilation – No. (%)	51 (40.5)
Ventilator-free days	28 (median)
APACHE II score	21 (median)
SOFA score	8 (median)
**Laboratory findings**	
C-reactive protein (mg/L)	122.5 (median)
Ferritin (μg/L)	573 (median)
Procalcitonin (μg**/**L)	0.5 (median)
D-dimer (mg/L)	1.83 (median)
Lactate (mmol/l)	1.22 (median)
**GSE156063**	
**Tissue resource**	Nasopharyngeal swabs
**Sample size**	n = 234 (93 COVID-19 patients versus 141 controls)
**Demographic characteristics**	
Male sex – No. (%)	110 (47.0)
Age Range – No. (%)	
Younger than 50	117 (50.0)
51–60	33 (14.1)
61–70	42 (17.9)
71–80	29 (12.4)
81 and older	13 (5.6)
**GSE152075**	
**Tissue resource**	Nasopharyngeal swabs
**Sample size**	n = 484 (430 COVID-19 patients versus 54 controls)
**Demographic characteristics**	
Male sex – No. (%)	200 (41.3)
Age Range – No. (%)	
Younger than 50	195 (40.3)
51–60	91 (18.8)
61–70	64 (13.2)
71–80	66 (13.6)
81 and older	51 (10.5)
Missing data	17 (3.5)

### Quantifying immune signatures’ enrichment levels

The single-sample gene-set enrichment analysis (ssGSEA) (19) was utilized to evaluate an immune signature’s enrichment level in a COVID-19 patient based on the expression profiles of its marker genes. The ssGSEA output the enrichment score of a gene set in a sample based on the degree of the genes in the gene set coordinately up- or down-regulated in the sample (19). The ratios of two immune signatures were defined as the base-2 log-transformed values of the geometric mean expression levels of all marker genes of an immune signature divided by those of another immune signature. The marker genes of the immune signatures analyzed were shown in **Table 2**.

**Table 2:** Immune signatures and their marker genes.

Immune signature	Marker genes
NK cells	*KLRC1, KLRF1*
Immune cytolytic activity	*PRF1, GZMA*
Th1 cells	*IFNG, TBX21, CTLA4, STAT4, CD38, IL12RB2, LTA, CSF2*
M1 macrophages	*FCGR1A, IDO1, SOCS1, CXCL10*
M2 macrophages	*MRC1, TGM2, FCER2, CCL22*
CD8+ T cells CD4+ regulatory T cells	*CD8A* *CTLA4, FOXP3, GPR15, IL32, IL4, IL5*
pro-inflammatory cytokines	*IFNG, IL-1A, IL-1B, IL-2*
anti-inflammatory cytokines PD-1	*IL-4, IL-10, IL-11, TGFB1* *PDCD1*
Type I IFN response	*DDX4, IFIT1, IFIT2, IFIT3, IRF7, ISG20, MX1, MX2, RSAD2, TNFSF10*

### Identifying DEGs between COVID-19 patients and normal people

We identified DEGs between COVID-19 patients and normal people by Student’s *t* test with a threshold of adjusted *P*-value < 0.05 and mean expression fold change (FC) > 1.5. The Benjamini-Hochberg method (20) was used to calculate the false discovery rate (FDR) to adjust for *P*-values in multiple tests.

### Clustering analysis

We hierarchically clustered COVID-19 patients based on the expression profiles of the common DEGs between COVID-19 patients and normal people in the three datasets.

Before clustering, we normalized gene expression values by z-score and transformed them into distance matrices by the R function “dist” with the parameter: method = “Euclidean.” The hierarchical clustering was performed with the function “hclust” in the R* *package *“*Stats” with the parameters: method = “ward.D2” and members = NULL.

### Statistical analysis and visualization

In comparisons of two classes of data, the Mann–Whitney *U* test was used if they were not normally distributed; otherwise, Student’s *t* test was utilized. The Pearson or Spearman method was employed to evaluate the correlation between two groups of data. The Fisher’s exact test was utilized to evaluate the association between two categorical variables. With the input of a significance level of 0.05, large effect sizes recommended by Cohen (21), and the sample sizes of COVID-19 patients and normal controls in the datasets into related functions in the R package “pwr“, we performed power calculations for all statistical tests in this study. All statistical analyses were performed in the R programming environment (version 4.1.2). The R package “ggplot2” was utilized to plot figures.

## Results

### Common DEGs between COVID-19 patients and normal people in the three datasets

This analysis found 13 genes whose expression levels were significantly higher in COVID-19 patients than in normal controls, consistently in the three datasets (two-tailed Student’s *t* test, FDR < 0.05; FC > 1.5). These genes included *OAS1*, *OAS2*, *OAS3*, *OASL*, *HERC6*, *SERPING1*, *IFI6*, *IFI44*, *IFI44L*, *CMPK2*, *RSAD2*, *EPSTI1*, and *CXCL10* (**Table 3**).

**Table 3:** The 13 genes significantly upregulated in COVID-19 patients.

Gene symbol	Gene ID	Full Name
***OAS1***	4938	2’-5’-oligoadenylate synthetase 1
***OAS2***	4939	2’-5’-oligoadenylate synthetase 2
***OAS3***	4940	2’-5’-oligoadenylate synthetase 3
***OASL***	8638	2’-5’-oligoadenylate synthetase like
***HERC6***	55008	HECT and RLD domain containing E3 ubiquitin protein ligase family member 6
***SERPING1***	710	serpin family G member 1
***IFI6***	2537	interferon alpha inducible protein 6
***IFI44***	10561	interferon induced protein 44
***IFI44L***	10964	interferon induced protein 44 like
***CMPK2***	129607	cytidine/uridine monophosphate kinase 2
***RSAD2***	91543	radical S-adenosyl methionine domain containing 2
***EPSTI1***	94240	epithelial stromal interaction 1
***CXCL10***	3627	C-X-C motif chemokine ligand 10

Notably, all these genes are involved in antiviral immune responses or immune regulation (22–30). Among these genes, four genes (*OAS1*, *OAS2*, *OAS3*, and *OASL*) encode members of the 2′–5′-oligoadenylate synthetase (OAS) family (31). It is justified that these genes are upregulated in COVID-19 patients since the OAS family plays important roles in the antiviral activity of interferons (32). *HERC6* (HECT And RLD Domain Containing E3 Ubiquitin Protein Ligase Family Member 6) is related to pathways of class I MHC mediated antigen processing and presentation and innate immune system (33, 34). *SERPING1* (serpin family G member 1) encodes a highly glycosylated plasma protein functioning in the regulation of the complement cascade (35). Several genes encode proteins induced by interferon, including *IFI6* (interferon alpha inducible protein 6), *IFI44* (interferon induced protein 44), and *IFI44L* (interferon induced protein 44 like). *CMPK2* (cytidine/uridine monophosphate kinase 2) encodes an enzyme in the nucleotide synthesis salvage pathway involved in the regulation of terminal differentiation of monocytic cells (36). *RSAD2* (radical S-adenosyl methionine domain containing 2) encodes a member of the S-adenosyl-L-methionine (SAM) superfamily of enzymes, which is an interferon-inducible antiviral protein playing a role in antiviral response and innate immune signaling (37). *EPSTI1* (epithelial stromal interaction 1) encodes a protein playing a role in M1 macrophage polarization (27). *CXCL10* (C-X-C motif chemokine ligand 10) is an antimicrobial gene encoding a chemokine of the CXC subfamily and ligand for the receptor CXCR3. The binding of CXCL10 to CXCR3 exerts pleiotropic effects on immune regulation (38). CXCL10 could also regulate the “cytokine storm” response to SARS-CoV-2 infection (39).

**Figure 1:**
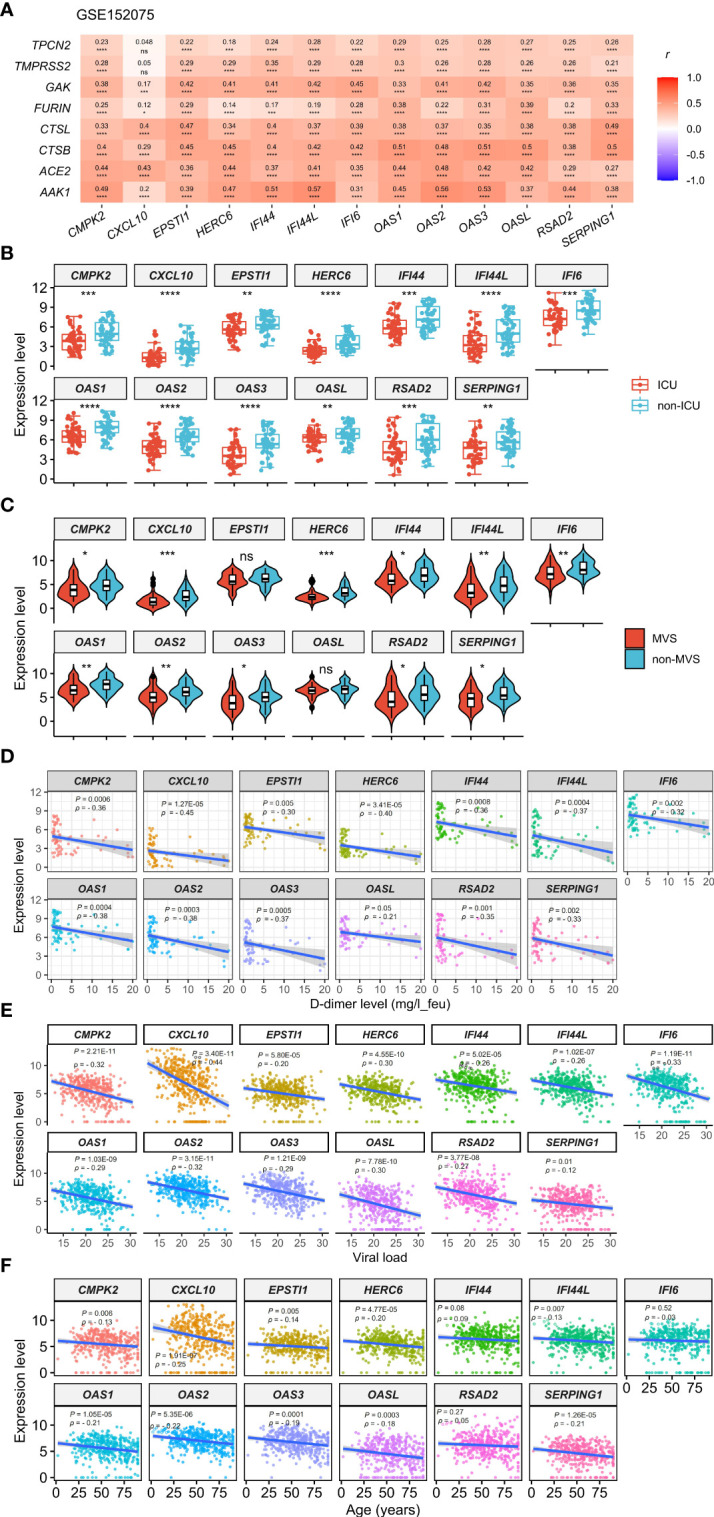
Expression correlations of the 13 genes upregulated in COVID-19 patients with the key regulators of SARS-CoV-2 infection and clinical features of COVID-19 patients. **(A)** Heatmap showing expression correlations between the 13 genes and 8 key regulators of SARS-CoV-2 infection in GSE152075. Pearson correlation coefficients (*r*) and *P*-values are shown. **(B, C)** Comparisons of the 13 genes’ expression levels between ICU and non-ICU, and between MVS and non-MVS COVID-19 patients. Two-tailed student’s *t* test *P*-values are shown. **(D–F)** Expression correlations of the 13 genes with D-dimer levels, viral loads, and ages of COVID-19 patients. Spearman correlation coefficients (*ρ*) and *P*-values are shown. The results shown in **(B–F)** were obtained by analyzing the dataset GSE157103. ICU, intensive care unit; MVS, mechanical ventilatory support. *P < 0.05, **P < 0.01, ***P < 0.001, ****P < 0.0001, ns: not significant. They also apply to the following figures.

This analysis found that the 13 upregulated genes likely had significant positive expression correlations with the genes which are key regulators of SARS-CoV-2 infection, including *ACE2*, *TMPRSS2*, *AAK1*, *CTSB*, *CTSL*, *FURIN*, *GAK*, and *TPCN2* (6–8, 40–43) (Pearson correlation, *P* < 0.05) (**Figure 1A**, **Supplementary Figure S1**). Interestingly, these genes were significantly downregulated in the intensive care unit (ICU) COVID-19 patients versus non-ICU patients (two-tailed Student’s *t* test, FDR < 0.05; FC > 1.5) in GSE157103 (**Figure 1B**). 11 of the 13 genes were significantly downregulated in the mechanical ventilatory support (MVS) patients versus non-MVS patients (FDR < 0.05; FC > 1.5) (**Figure 1C**). Moreover, all these genes showed significant negative expression correlations with D-dimer levels (Spearman correlation, FDR < 0.05) (**Figure 1D**), whose elevation was associated with COVID-19 severity (44). In GSE152075, the expression levels of the 13 genes were significantly and negatively correlated with viral loads (Spearman correlation, FDR < 0.05) (**Figure 1E**). In addition, this analysis found that many of the 13 genes had significant negative expression correlations with ages of COVID-19 patients (**Figure 1F**). Taken together, these results indicate that upregulation of these molecules is associated with better clinical outcomes in COVID-19 patients.

### Identification of COVID-19 subtypes based on expression profiles of the 13 upregulated genes in COVID-19 patients

**Figure 2:**
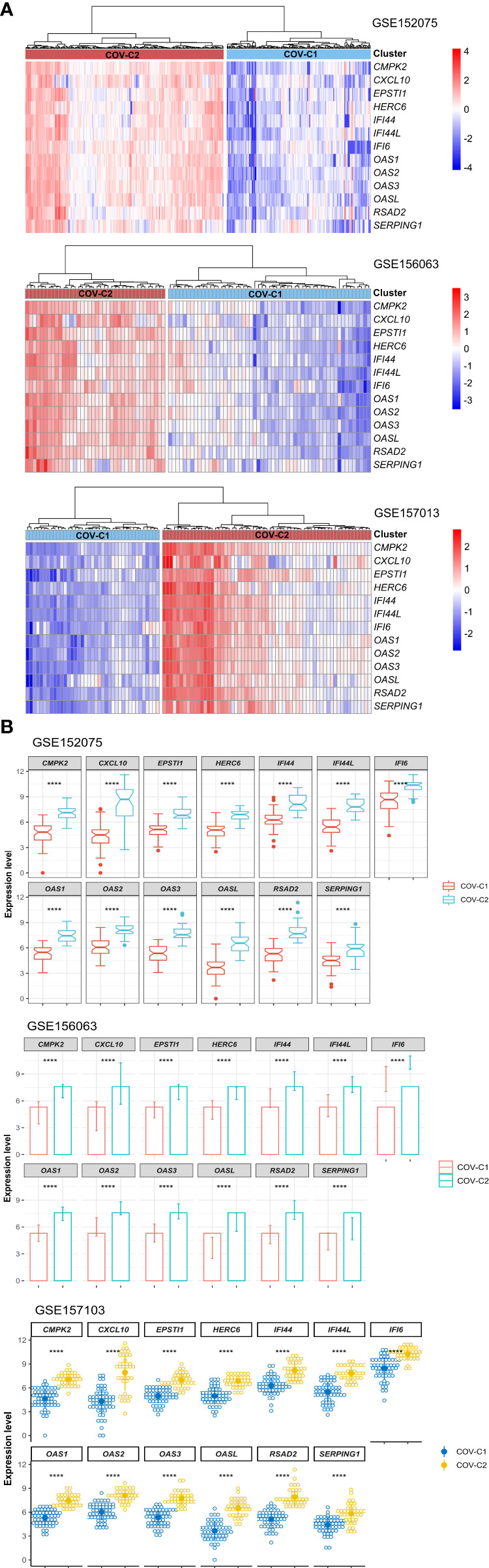
Identification of COVID-19 subtypes based on expression profiles of the 13 upregulated genes in COVID-19 patients. **(A)** Hierarchical clustering identifying two COVID-19 subtypes: COV-C1 and COV-C2, consistently in three datasets. **(B)** The 13 genes have significantly higher expression levels in COV-C2 than in COV-C1. Two-tailed student’s *t* test *P*-values are shown. ****P < 0.0001.

**Figure 3:**
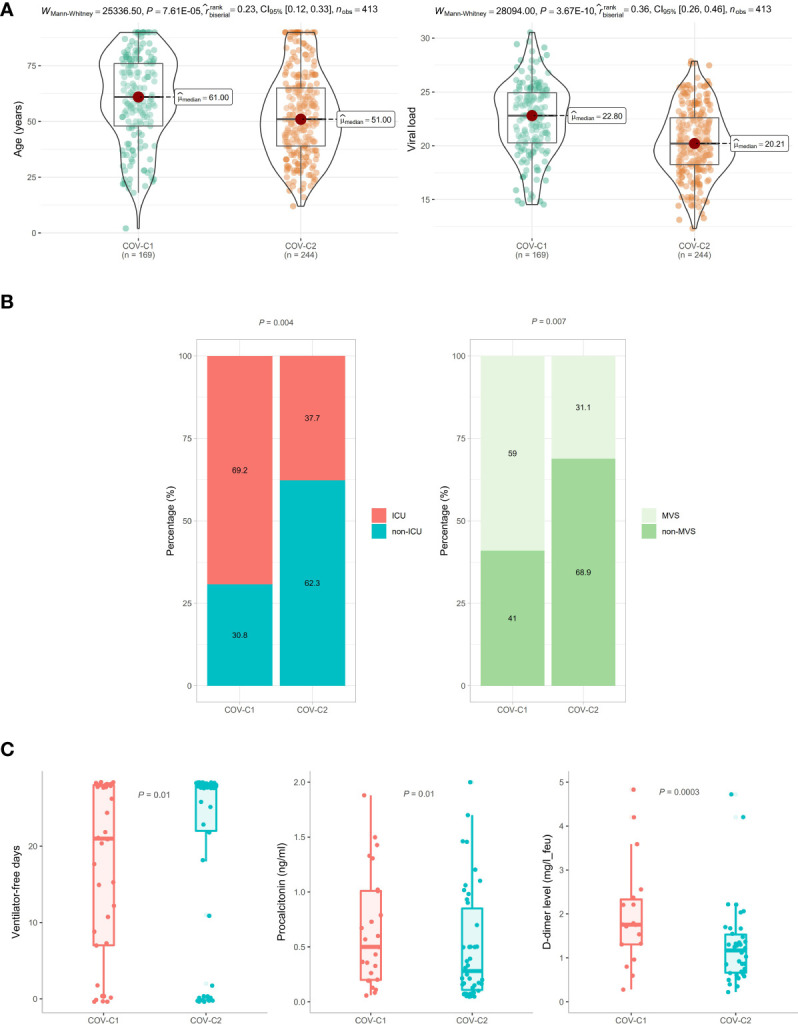
Comparisons of clinical features between COV-C1 and COV-C2 patients. **(A)** In GSE152075, COV-C2 patients are younger and have significantly lower viral loads than COV-C1 patients. **(B)** COV-C2 has significantly lower proportions of ICU patients and MVS patients than COV-C1. Fisher’s exact test *P*-values are shown. **(C)** COV-C2 patients are with significantly more ventilator-free days and have significantly lower levels of D-dimer and procalcitonin than COV-C1 patients. One-tailed Mann–Whitney *U* test *P*-values are shown. The results shown in **(B, C)** were obtained by analyzing the dataset GSE157103.

Based on the expression levels of the 13 genes upregulated in COVID-19 patients, we identified two COVID-19 clusters consistently in the three datasets (GSE152075, GSE156063, and GSE157103) by hierarchical clustering. We termed them COV-C1 and COV-C2, respectively (**Figure 2A**). The 13 genes had significantly higher expression levels in COV-C2 than in COV-C1 (FDR < 0.05; FC > 1.5) (**Figure 2B**). In GSE152075, COV-C2 patients were younger than COV-C1 patients (one-tailed Mann–Whitney *U* test, *P* = 7.61 × 10^-5^), while COV-C2 had significantly lower viral loads than COV-C1 (*P* = 3.67 × 10^-10^) (**Figure 3A**). It indicates a significantly negative association between age and viral loads, in line with a previous report (45). In GSE157103, the proportion of ICU patients was significantly lower in COV-C2 than in COV-C1 (37.7% versus 69.3%; Fisher’s exact test, *P* = 0.004); the proportion of MVS patients was also significantly lower in COV-C2 than in COV-C1 (31.1% versus 59.0%; Fisher’s exact test, *P* = 0.007) (**Figure 3B**). Ventilator-free days is a measure of outcomes in treating acute respiratory distress syndrome and correlate inversely with disease severity (46). This analysis found that COV-C2 patients were with significantly more ventilator-free days than COV-C1 patients (*P* = 0.01) (**Figure 3C**). In addition, several COVID-19 severity-associated laboratory measurements, such as D-dimer and procalcitonin, displayed significantly lower levels in COV-C2 than in COV-C1 (*P* < 0.02) (**Figure 3C**). Altogether, these results indicated better outcomes in COV-C2 compared with COV-C1.

### COV-C2 patients have significantly stronger antiviral immune signatures than COV-C1 patients

**Figure 4:**
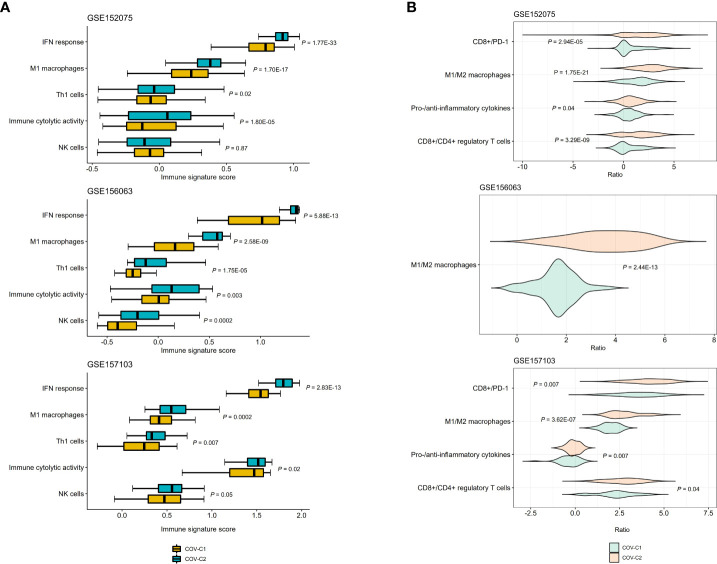
Comparisons of antiviral immune signatures between COV-C1 and COV-C2 patients. **(A)** COV-C2 patients likely have higher enrichment scores of antiviral immune signatures than COV-C1 patients. One-tailed Mann–Whitney *U* test *P*-values are shown. **(B)** COV-C2 patients have significantly higher ratios of immunostimulatory over immunoinhibitory signatures. Two-tailed student’s *t* test *P*-values are shown.

We compared the enrichment scores (ssGSEA scores) of five antiviral immune signatures between COV-C1 and COV-C2. These immune signatures included NK cells, immune cytolytic activity, type I interferon (IFN) response, M1 macrophages, and Th1 cells. This analysis found that the enrichment scores of these immune signatures were likely higher in COV-C2 than in COV-C1 (**Figure 4A**). Moreover, the ratios of immunostimulatory/immunoinhibitory signatures (CD8+/CD4+ regulatory T cells, M1/M2 macrophages, pro-/anti-inflammatory cytokines, and CD8+/PD-1) were likely higher in COV-C2 than in COV-C1 (**Figure 4B**). Altogether, these results suggested the stronger antiviral immune responses displayed in COV-C2 versus COV-C1 patients. The stronger antiviral immune responses could contribute to better outcomes in COV-C2 relative to COV-C1 patients.

## Discussion

In this study, we identified 13 genes significantly upregulated in COVID-19 patients’ leukocyte and SARS-CoV-2-infected nasopharyngeal tissue. This analysis found that all these genes are involved in the regulation of host immune signaling. Moreover, these genes’ upregulation is likely associated with better clinical outcomes in COVID-19 patients. It is justified since these molecules’ upregulation is associated with increased antiviral immune responses to reduce COVID-19 disease severity. Based on the expression profiles of the 13 genes, we uncovered two COVID-19 subtypes (COV-C1 and COV-C2) reproducibly in three different datasets. Compared with COV-C1, COV-C2 more highly expressed these upregulated genes in COVID-19 patients and had stronger antiviral immune responses. As a result, COV-C2 displayed more favorable clinical outcomes.

The expression levels of the 13 genes were likely to correlate negatively with ages of COVID-19 patients, and COV-C1 patients were younger than COV-C1 patients. These results suggest that younger patients have stronger antiviral immune responses than old patients. It could partially explain why younger patients are less likely to develop severe COVID-19 (9). Intriguingly, younger patients had lower viral loads than old patients, although the former displayed stronger antiviral immune responses than the latter. It is reasonable since the stronger antiviral immune responses promote the elimination of more SARS-CoV-2 viruses in younger patients.

Our results showed no significant association between sex and antiviral immune responses in COVID-19 patients, although female patients are likely to have better prognosis than male patients (10). In fact, some other factors could be responsible for the better outcomes in female versus male patients, such as estrogen levels (47), C-reactive protein abundance (48), lifestyle (49), underlying conditions (50), and renin-angiotensin system  (51).

Our results suggest that a strong antiviral immune response is essential in reducing severity of COVID-19. However, excessive immune response, known as “cytokine storm,” may result in worse clinical outcomes in COVID-19 patients (52). These results collectively indicate that both insufficient and excessive immune responses may contribute to COVID-19 severity.

This study has several limitations. First, this study obtained the results merely by bioinformatics analyses, whose reliability needs to be validated by experimental and clinical data. Second, because the sample size in this study is limited, the results need to be supported by analyzing more datasets. Nevertheless, the power analysis showed that all the powers of the statistical tests were greater than 0.95 using a significance threshold of 0.05, large effect sizes, and the sample sizes of the datasets. Finally, the results and conclusions were obtained by analyzing mRNA expression data. However, the gene expression pattern is not necessarily identical to the protein expression pattern due to some factors influencing the mapping from gene expression to protein expression, e.g., post-translational modification. Thus, the verification of our findings at protein level is a must.

## Data availability statement

The original contributions presented in the study are included in the article/**Supplementary Material**. Further inquiries can be directed to the corresponding authors.

## Ethics statement

Ethical review and approval was not required for the study on human participants in accordance with the local legislation and institutional requirements. The patients/participants provided their written informed consent to participate in this study.

## Author contributions

ZD: software, validation, formal analysis, investigation, data curation, visualization, writing-review & editing. QY: Software, validation, formal analysis, visualization. WC: software, validation, investigation. ZL: investigation, supervision, funding acquisition. XW: conceptualization, methodology, resources, investigation, writing-original draft, writing-review & editing, supervision, project administration, funding acquisition. All authors contributed to the article and approved the submitted version.

## Funding

This work was supported by the China Pharmaceutical University (grant number 3150120001 to XW), Natural Science Foundation of Jiangsu Province (grant number BK20201090 to ZL), and China Postdoctoral Science Foundation (grant number 2021M691338 to ZL).

## Conflict of interest

The authors declare that the research was conducted in the absence of any commercial or financial relationships that could be construed as a potential conflict of interest.

## Publisher’s note

All claims expressed in this article are solely those of the authors and do not necessarily represent those of their affiliated organizations, or those of the publisher, the editors and the reviewers. Any product that may be evaluated in this article, or claim that may be made by its manufacturer, is not guaranteed or endorsed by the publisher.

## Supplementary material

Supplementary Figure 1 Heatmap showing expression correlations between the 13 genes and 8 key regulators of SARS-CoV-2 infection in GSE157103 and GSE156063. Pearson correlation coefficients (*r*) and *P*-values are shown.

Supplementary Table 1 The details on the datasets used in this study.
