# Supplementary figures and images for: Identification of key molecules in COVID-19 patients significantly correlated with clinical outcomes by analyzing transcriptomic data
Source: Front Immunol. 2022 Aug 22;13:930866. doi: 10.3389/fimmu.2022.930866 (PMC9441550; doi:10.3389/fimmu.2022.930866)

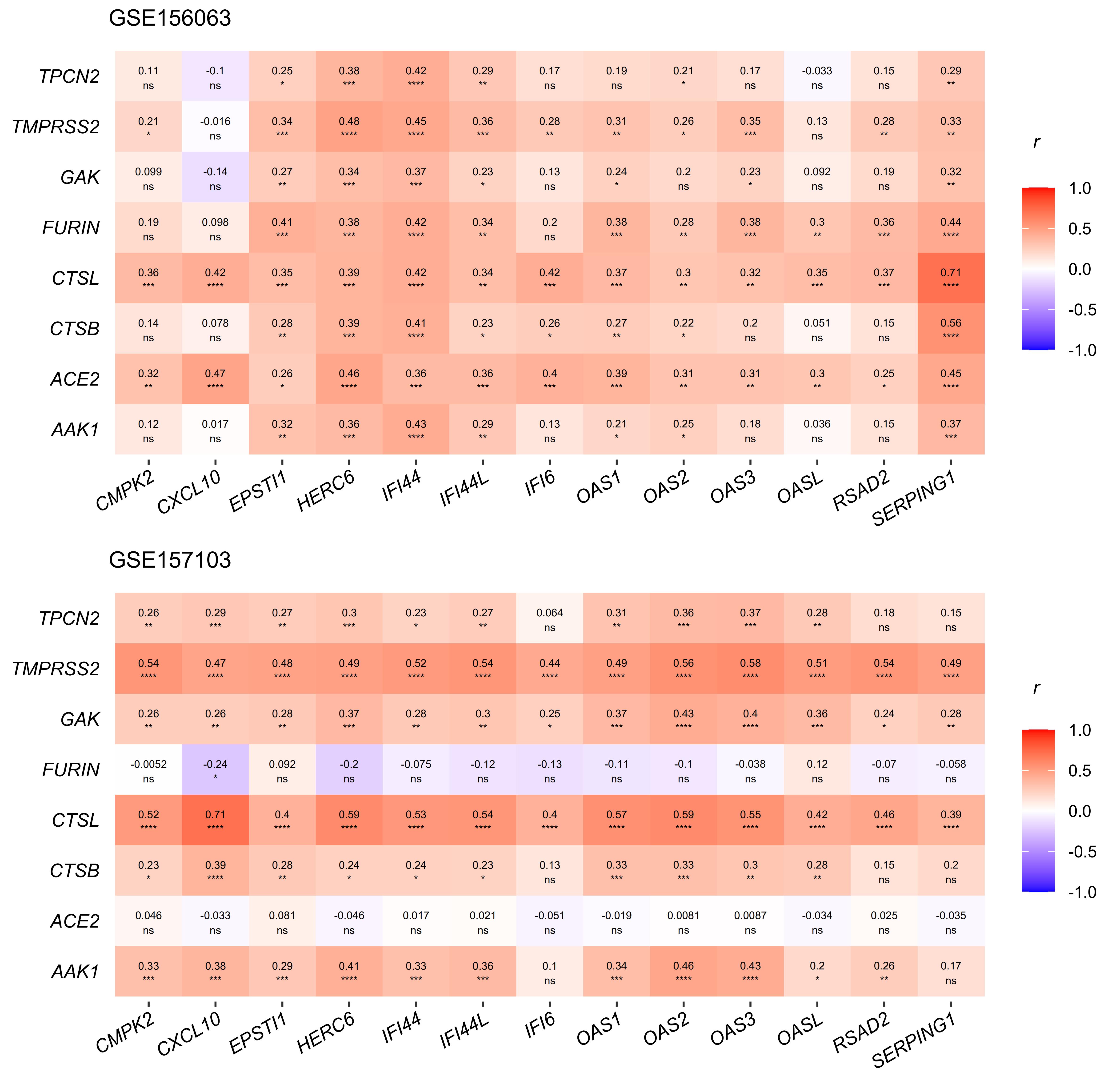

Supplement: Supplementary Figure 1 — Heatmap showing expression correlations between the 13 genes and 8 key regulators of SARS-CoV-2 infection in GSE157103 and GSE156063. Pearson correlation coefficients (r) and P-values are shown. [file Image_1.jpeg]
